# Supplementary material for: Effect of graphene addition on tensile, flexural, and hardness behavior of GFRP composites
Source: PLoS One. 2026 Jan 23;21(1):e0340894. doi: 10.1371/journal.pone.0340894 (PMC12829930; doi:10.1371/journal.pone.0340894)
Supplement: Attachment — Submitted filename: Responses to Reviewers.docx [file pone.0340894.s003.docx]

**Response to the Editor and Reviewers**

**Manuscript ID:** PONE-D-25-37781R1

**Title:** *Effect of Graphene Addition on Tensile, Flexural, and Hardness Behavior of GFRP Composites*

Dear Editor and Reviewers,

We sincerely thank the Editor and Reviewers for their valuable comments and suggestions, which have helped us improve the quality and clarity of our manuscript. We have carefully revised the manuscript in accordance with the comments. All changes have been incorporated in the revised version and highlighted using the Track Changes mode. Our detailed, point-by-point responses are provided below.

**Editorial and Technical Corrections**

**Editor’s comments**

Journal Requirements:

When submitting your revision, we need you to address these additional requirements.

**Comment #1**

**#1.** We note you have included a table to which you do not refer in the text of your manuscript. Please ensure that you refer to Table 1, 2, and 3 in your text; if accepted, production will need this reference to link the reader to the Table.

**Reply**

All tables (Table 1, Table 2, and Table 3) are now appropriately cited and referred to within the main text of the manuscript to ensure clarity and proper linkage.

**Comment #2**

**#2.** We note that your author list was updated during the revision process. In order to add or remove authors or update the order of the author by-line after initial submission, we ask that authors complete an Authorship Change Request form.

**Reply**

The Authorship Change Request form has been duly completed and submitted as per the journal’s authorship policy.

**Comment 1**

1. Please ensure that your manuscript meets PLOS ONE's style requirements, including those for file naming.

**Responses**

Thank you for your guidance and to give attention to the journal requirements.

The manuscript has been reformatted according to PLOS ONE’s style and structure guidelines. File names, headings, references, figure captions, and table formats now comply with the PLOS ONE formatting requirements

Dear editor according to your suggestion we have included it in the revised manuscript, a rebuttal letter that responds to each point raised by the academic editor and reviewer(s). The correspondence author uploaded this letter as a separate file labeled **'Response to Reviewers'.**

- And also a marked-up copy of our revised manuscript that highlights changes made to the original version have been submitted to this as a separate file labeled **'Revised Manuscript with Track Changes'**.
- Again the unmarked version of our revised paper without tracked changes is also attached to this as a separate file labeled '**Manuscript'**.
- If you would like to make changes to your financial disclosure, please include your updated statement in your cover letter. Guidelines for resubmitting your figure files are available below the reviewer comments at the end of this letter, and also we filled the Author Change Form according to the Editor’ authorship format and journals guideline and submitted in online submission portal with this revised manuscript labeling as ’ **Other’**

**Responses**

**Dear editor thanks you very much for giving us the direction to write** financial disclosure. According to your suggestion we have included the APC waiver letter in the cover letter to indicate the financial disclosure.

**Comment 2**

2. In the online submission form you indicate that your data is not available for proprietary reasons and have provided a contact point for accessing this data. Please note that your current contact point is a co-author on this manuscript. According to our Data Policy, the contact point must not be an author on the manuscript and must be an institutional contact, ideally not an individual. Please revise your data statement to a non-author institutional point of contact, such as a data access or ethics committee, and send this to us via return email. Please also include contact information for the third party organization, and please include the full citation of where the data can be found.

**Response:**
Thank you for highlighting this issue. We have revised the Data Availability Statement to replace the author-based contact point with an institutional contact that is independent of all manuscript authors.

**Revised Data Availability Statement:**
All data underlying the findings of this study are fully available within the manuscript and its Supporting Information files. Due to institutional restrictions and confidentiality agreements, additional raw data cannot be publicly deposited. Data requests may be submitted to the Research Ethics office and Data Access Center at the Centre of Studies in Oil and Gas Engineering and Technology (CS-OGET), Eduardo Mondlane University, Maputo, Mozambique **(email: csoget.geral@uem.mz),** subject to ethical review and approval. We have update and tick the **“NO– some restrictions will apply” in the online submission system as well in this point by point response.**

**Reflections:**
The above contact represents an institutional research office responsible for data governance, ethics, and research integrity at Eduardo Mondlane University. This contact point ensures independent oversight and compliance with the university’s confidentiality and ethical standards.

Now this revision aligns fully with the PLOS ONE Data Policy requirements. Thank you again for your time, careful consideration, and support throughout this process.

**Comment 3**

3. When completing the data availability statement of the submission form, you indicated that you will make your data available on acceptance. We strongly recommend all authors decide on a data sharing plan before acceptance, as the process can be lengthy and hold up publication timelines. Please note that, though access restrictions are acceptable now, your entire data will need to be made freely accessible if your manuscript is accepted for publication. This policy applies to all data except where public deposition would breach compliance with the protocol approved by your research ethics board. If you are unable to adhere to our open data policy, please kindly revise your statement to explain your reasoning and we will seek the editor's input on an exemption. Please be assured that, once you have provided your new statement, the assessment of your exemption will not hold up the peer review process.

**Response to the Editor regarding Data Sharing Policy and Availability Statement**

**Dear Editor,**

Thank you very much for your detailed feedback and clarification regarding the data sharing requirements. We greatly appreciate your guidance in ensuring full compliance with the PLOS ONE Data Policy. Please find our response below.
Thank you for this important clarification. Due to institutional restrictions and confidentiality agreements established with the Centre of Studies in Oil and Gas Engineering and Technology (CS-OGET), Eduardo Mondlane University, the underlying raw data cannot be publicly shared in an open repository. These restrictions are based on confidentiality clauses and ethical compliance requirements that protect proprietary and participant-sensitive information related to ongoing institutional research projects.

**Revised Data Availability Statement**
All data underlying the findings of this study are fully available within the manuscript and its Supporting Information files.
Due to institutional restrictions and confidentiality agreements, additional raw data cannot be publicly deposited. Data requests may be submitted to the Research Ethics office and Data Access Center at the Centre of Studies in Oil and Gas Engineering and Technology (CS-OGET), Eduardo Mondlane University, Maputo, Mozambique **(email: csoget.geral@uem.mz),** subject to ethical review and approval. We have updated and tick the “**NO– some restrictions will apply” in the online submission system as well in this point by point response.**

We trust this explanation satisfies the journal’s requirements for data transparency while respecting the ethical and institutional boundaries under which the study was conducted

**Comment 4**

4. Please ensure that you refer to Figure 9 in your text as, if accepted, production will need this reference to link the reader to the figure.

**Response:** We have now added a reference to *Figure 9* in the manuscript text. The figure is discussed in the results section where the related data are interpreted.

**Comment 5**

5. We note you have included a table to which you do not refer in the text of your manuscript. Please ensure that you refer to Table 1, 2, and 3 in your text; if accepted, production will need this reference to link the reader to the Table.

**Response:** All tables (Table 1, Table 2, and Table 3) are now appropriately cited and referred to within the main text of the manuscript to ensure clarity and proper linkage.

**Comment 6**

6. If the reviewer comments include a recommendation to cite specific previously published works, please review and evaluate these publications to determine whether they are relevant and should be cited.

**Response:** We have reviewed the recommended citations from the previous review round. Relevant and recent studies on graphene-based GFRP composites have been added to the Introduction and Discussion sections to strengthen the literature background and support our findings.

**Comment 7**

**7. Data Availability Concern (Reviewer #2):** Reviewer #2 noted that the data availability was not clear.

**Response:** All data underlying the findings of this study are fully available within the manuscript and its Supporting Information files.

**Comment 8**

8. Please ensure that the manuscript is clearly written in standard English.

**Response:** The entire manuscript has been carefully proofread and language-edited for clarity, grammar, and conciseness.

# Response to Reviewer 1 Comments

## 1. Authors need to add more quantitative results in the abstract section.

**Author Response**: As per the reviewer’s suggestions, more quantitative results were added in the abstract section. The experimental results revealed an increase in tensile strength by 5.48 %, flexural strength by 27.74 %, and a reduction in micro hardness by 20 % for 4 wt. % filler added GFRP compared to neat GFRP, indicating reduced brittleness and enhanced ductility. Furthermore, addition of graphene resulted in a 9 % increase in elongation and improved interfacial bonding between glass fiber and epoxy matrix was observed. Scanning electron microscopy (SEM) analysis confirmed uniform graphene dispersion, reduced voids, and improved fiber–matrix adhesion. The results demonstrate that the inclusion of 4 wt. % graphene filler achieves an optimal balance between strength, flexibility, and interfacial bonding, making these composites suitable for structural and lightweight applications.

## 2. Consider explicitly stating the novelty of the current work toward the end of the Introduction.

Author Response: Author welcome the reviewer’s comments for giving suggestions on stating the novelty of the current work. The revised manuscript now concludes the Introduction part with a clear statement of novelty. The novelty of the present research lies in selection of correct combination of graphene filler concentration to achieve a balance between improved tensile and flexural properties. Some of the previous studies that focused solely on mechanical enhancement, this work emphasizes the interfacial microstructural improvements achieved through a controlled dispersion process involving thermal-assisted sonication and vacuum compaction. The systematic comparison of 0 wt. %, 2 wt. %, and 4 wt. % graphene filler content provides new insights into the synergistic effects of nanoparticle reinforcement on load transfer efficiency, ductility, and void minimization. The resulting composite formulation offers a cost-effective and scalable approach for developing next-generation lightweight structural materials.

## 3. wt. % can be written as wt. % (give a space).

This correction has been implemented consistently throughout the manuscript. All occurrences of 'wt.%' have been replaced with 'wt. %' to comply with formatting conventions.

## 4. How was the uniform distribution of Graphene powder in matrix phase ensured?

For the graphene added composites, a combination of mechanical stirring, thermal pre-treatment, and ultrasonic sonication was employed to ensure the uniform dispersion of graphene powder within the epoxy matrix. Initially, the graphene powder was added to the pre-heated epoxy resin at 70 °C to facilitate wetting and breakdown of agglomerates. The mixture was allowed to cool at room temperature at a slower rate by partially heating it. Sonication was performed for about 20 minutes at 150 V; then, the hardener was added, and stirring continued with the mechanical stirrer for another 5minutes. This dual approach of sonication and thermal mixing promoted homogeneous distribution of graphene nanosheets throughout the epoxy matrix, minimizing particle agglomeration and enhancing filler–matrix interaction. The uniform dispersion was visually confirmed by the consistent surface morphology of the prepared composites and further validated through SEM analysis, which revealed minimal clustering and improved interfacial bonding between the graphene particles and polymer matrix.

## Reviewer Comment: 5. Paper requires considerable modification. The manuscript contains few typos which should be corrected. Some words are merged. Check the text for clarity, grammar and syntax throughout.

## 400x400 MM …………. MM must be written in small letter

## • 70°C, 190.33N............. give a space between value and unit, and do similar corrections throughout the manuscript.

## • GFRP with 4% filler material…. Mention wt. % or vol. %

As per reviewer comments, thorough proofreading of the entire manuscript has been done. Typographical errors, merged words, and spacing issues have been corrected. 'MM' has been changed to 'mm', spaces were added between values and units (e.g., 70 °C, 190.33 N), and filler content has been uniformly written as 'wt. %'. Grammar and syntax have also been refined throughout.

## 6. Add more suitable references for the mentioned ASTM standards. Authors can cite following article.

## ASTM D3039: Exploring the use of TiO2 filler in short Ficus Benghalensis natural fibre-based polymeric composites. ASTM D790: Mechanical and tribo-performance analysis of LD sludge filled wood apple dust-epoxy composites using response surface methodology. ASTM E384: TRIBOLOGICAL STUDY ON SLURRY ABRASIVE WEAR BEHAVIOR OF NONWOVEN VISCOSE FABRIC COMPOSITES WITH DOE APPROACH

Author Response: The suggested references for ASTM D3039 [41], ASTM D790 [42], and ASTM E384 [43] have been included in the Materials and Methods section to strengthen the experimental methodology and demonstrate alignment with contemporary studies. The recommended articles are included in reference section.

## 7. In Fig 12, annotate SEM images to make it easier for readers to connect what you described in text with the visual.

Author Response: Figure 12 (New Figure No 13) has been revised with proper annotations highlighting fractured fiber, delamination,voids, glass fiber, graphene flakes, aligned fibers, agglomerates, and aligned fibers . Annotation was done according the description given in the text.

## 8. How the content of filler selected?

Author Response: We appreciate the reviewer’s insightful query. The selection of filler content was based on previous studies and preliminary experimental trials. Reported literature on graphene-reinforced epoxy and glass fiber composites indicates that filler loadings below 1 wt. % produce minimal reinforcement effects, while higher loadings above 5 wt. % often lead to agglomeration, poor dispersion, and reduced interfacial bonding strength [13, 19]. Therefore, graphene contents of 2 wt. % and 4 wt. % were chosen in this study to achieve effective dispersion, enhanced fiber–matrix adhesion, and measurable improvement in mechanical properties without compromising processability.

## 9. Previous research on glass fiber and particulate filler needs to be described in detail to obtain research gaps so that the research position becomes clear and this topic is worthy of research. Authors can cite recent papers like; Mechanical and tribological characteristics of glass fiber and rice stubble-filled epoxy-LD sludge hybrid composites. Mechanical and Tribo-performance analysis of Linz Donawitz sludge-filled glass–epoxy composites using Taguchi experimental design.

Author Response: We appreciate the author suggestions. In the Introduction recent studies on glass fiber and particulate-filled composites, including the two suggested papers has been included to highlight the research gap. Recent investigations on hybrid glass fiber composites reinforced with particulate fillers have demonstrated noticeable improvements in mechanical and tribological behavior. For instance, Mechanical and tribological characteristics of glass fiber and rice stubble-filled epoxy–LD sludge hybrid composites reported that hybrid fillers enhanced wear resistance and load-bearing capacity through improved interfacial bonding [39]. Similarly, Mechanical and tribo-performance analysis of Linz Donawitz sludge-filled glass–epoxy composites using Taguchi experimental design highlighted the influence of optimized filler ratios on flexural strength and wear performance [40]. However, despite these promising developments, most studies have focused on micro-scale or waste-derived fillers, and limited attention has been given to the effect of graphene nanoparticle reinforcement on the mechanical and interfacial characteristics of GFRP composites.

## 10. Add technical facts to the conclusions to improve them.

Author Response: The Conclusions section has been revised to include more quantitative and technically supported statements, specifying tensile, flexural, and hardness improvements along with corresponding microstructural observations.

The present study investigated the influence of graphene powder addition on the mechanical and microstructural behavior of Glass Fiber Reinforced Polymer (GFRP) composites fabricated using the hand lay-up method followed by vacuum bagging. The experimental results demonstrated that the inclusion of graphene significantly enhanced the composite performance depending on the filler content. The 4 wt. % graphene-added composite showed the maximum improvement in tensile and flexural behavior, with an increase of 5.48% in tensile strength and 27.74 % in flexural strength compared to the plain GFRP composite. These improvements are primarily attributed to enhanced interfacial adhesion between the glass fibers and epoxy matrix, as confirmed by SEM analysis, which revealed uniform graphene dispersion, fewer voids, and restricted fiber pullout. Furthermore, the microhardness decreased by 20% with the addition of 4 wt. % graphene, indicating reduced brittleness and improved ductility of the composite. This reduction in surface hardness suggests that graphene facilitates better energy absorption under deformation, which is beneficial for applications requiring impact resistance.

## 11. In conclusion, be more specific in applications.

Author Response: As per the reviewer suggestions, the Conclusions was modified to specify practical applications of the developed composites. The enhanced graphene-modified GFRP composites are well-suited for aerospace structures, automotive body panels, wind turbine blades, marine applications, and sports equipment, where a high strength-to-weight ratio, durability, and improved fatigue resistance are essential. The study also provides a foundation for future research on optimizing nanofiller dispersion techniques, scaling the fabrication process for industrial applications, and exploring hybrid filler systems to further tailor the multifunctional properties of advanced composite materials.

# Response to Reviewer 2 Comments

**The manuscript is well written, and the results well presented. However, by adding fillers and claiming that the hardness decreases and therefore the ductility increases, it is essential to investigate the stress-strain curves to see if a yield point can be observed in the specimen. I would, therefore, like to see these curves either in the manuscript or separately provided for me by the editor.**

Author response: We sincerely thank the reviewer for this insightful comment. Using the experimental data tensile stress–strain curves for the 0 wt. %, 2 wt. %, and 4 wt. % graphene–GFRP composites and added in the manuscript results and discussion 3.1 section.

Figure 7. Stress strain curve of the different filler added Composites

The tensile stress–strain diagram of the GFRP composites with varying graphene content (0 wt. %, 2 wt. %, and 4 wt. %) are shown in Figure 7. GFRP specimens exhibits by an initial linear-elastic region followed by a gradual deviation from linearity prior to failure. The plain composite (0 wt. %) shows a predominantly linear response up to the ultimate stress, indicating a brittle failure mode governed by fibre fracture. In contrast, the 2 wt. % and 4 wt. % graphene-filled composites show a noticeable nonlinear region before the ultimate tensile strength (UTS), indicating progressive matrix yielding, fibre–matrix debonding, prior to rupture. Although a distinct yield point, as observed in metallic materials, is not present in fibre-reinforced polymers, the onset of nonlinearity in the 2 wt. % and 4 wt. % composites signifies a higher strain tolerance and delayed catastrophic failure. This behavior leads to a slight reduction in hardness corresponds to improved deformability of the matrix and enhanced ductility of the composite system. The inclusion of graphene improves interfacial adhesion and energy dissipation capability, allowing limited plastic deformation before complete failure. The stress–strain curves thus confirm the ductility enhancement associated with graphene reinforcement while maintaining the strength characteristics of the GFRP composites.

# Response to Reviewer 3 Comments

**1. How was the repeatability of each test ensured, and were standard deviations or error bars considered in the reported data?**

Author response: Each mechanical test (tensile, flexural, and hardness) was repeated three times for every composition (0 wt.%, 2 wt.%, and 4 wt.% graphene) to ensure repeatability. The reported values in the revised manuscript represent the average of three independent trials, and the variation between trials was within ±3 %.

**2. What calibration procedure was followed for the instruments used to maintain measurement accuracy throughout the experiments?**

Author response: All instruments were calibrated prior to testing. The Mecmesin Multi-Tester was calibrated using certified load cells (0–25 kN) following the manufacturer’s calibration routine. The Matsuzawa micro-hardness tester (Model MMT-X7A) was verified with a standard reference block of 100 HV before each test batch. These calibrations ensured measurement precision and reproducibility.

**3. Were environmental conditions such as temperature and humidity controlled during testing, and could they influence the observed outcomes?**

Author response: All mechanical tests were performed under controlled laboratory conditions maintained at 25 ± 2 °C and 50 ± 5 % relative humidity. These controlled conditions minimized the influence of environmental factors on the epoxy matrix and ensured consistency across specimens.

**4. What is the rationale behind the selected process parameters or test conditions—were they optimized or based on preliminary trials or literature data?**

Author response: The parameters (graphene contents of 0 wt. %, 2 wt. %, and 4 wt. %) were selected based on preliminary trials and supporting literature. Lower concentrations (< 1 wt.%) showed negligible improvements, while higher loadings (> 5 wt.%) caused graphene agglomeration and poor dispersion. The applied testing parameters (loading rate = 2 mm/min, span length = 110 mm) followed ASTM D3039 and D790 standards.

**5. How was the sample preparation process standardized to avoid variation between different test specimens?**

Author response: All specimens were fabricated under identical processing conditions using the hand lay-up method followed by vacuum bagging. The graphene dispersion process was standardized through a combination of mechanical stirring, controlled thermal mixing at 70 °C, and 20 min ultra-sonication, ensuring uniform filler distribution. The glass-fiber reinforcement ratio was maintained constant at 60 wt. % for every batch.

**6. The paper reports improved results under specific conditions. What are the underlying microstructural or physicochemical mechanisms responsible for this improvement?**

Author response: The improved mechanical performance is attributed to enhanced interfacial bonding and uniform graphene dispersion, which reduced voids and improved stress transfer efficiency. SEM analysis (Figure 12) revealed better matrix continuity and fewer fiber pull-outs, confirming Graphene role in bridging the matrix–fiber interface and enhancing load transfer.

**7. Were any anomalies or outliers observed in the dataset, and how were they treated during analysis?**

Author response: No such anomalies or outliers observed in the dataset.

**8. Have the authors considered the influence of interfacial bonding or phase interaction in explaining the observed property enhancement?**

Author response: Yes. Interfacial bonding and phase interaction were discussed in Sections 3.1 and 3.4 of the revised manuscript. Graphene improved epoxy–fiber adhesion through increased surface energy and chemical compatibility, promoting better load sharing and delayed crack propagation.

**9. Can the authors provide a mechanistic explanation linking the morphology or microstructure to the obtained performance outcomes?**

Author response: We thank the reviewer for this valuable comment. The mechanistic explanation was included in Section 3.4 SEM characterization. The morphology and microstructural features of the fabricated composites were closely correlated with their mechanical performance. The scanning electron microscopy (SEM) analysis revealed distinct morphological differences between the unfilled and graphene-filled GFRP composites. For the 0 wt. % composite, micrographs show interfacial gaps, fiber pull-outs, and matrix cracking, indicating poor stress transfer and premature interfacial failure. In contrast, the 2 wt. % and 4 wt. % graphene-filled composites exhibit uniform filler dispersion within the epoxy matrix and improved fiber–matrix adhesion. The graphene Nano sheets act as micro-bridges at the interface, improving load transfer efficiency and delaying crack initiation. At higher graphene loading (4 wt. %), the network of graphene platelets reinforces the matrix by restricting polymer chain mobility and hindering crack propagation through deflection and branching. These microstructural mechanisms explain the increase in tensile and flexural strength and the slight decrease in hardness, which collectively indicate a transition toward a more ductile failure mode. The uniform Graphene distribution also facilitates energy dissipation during deformation, contributing to enhanced toughness and strain accommodation.

**10. How would the results change if the testing was performed under dynamic or real operating conditions instead of static conditions?**

Author response: Under dynamic or cyclic loading, the improved interfacial bonding achieved with graphene reinforcement is expected to yield enhanced fatigue and impact resistance. Future work will focus on dynamic loading experiments to evaluate the composites’ performance under real operating conditions, such as vibration and impact fatigue.

**11. The following references are recommended for citation. Soundararajan, R., Dharunprakash, E., Arjunkumaar, N. et al. Appraisal of Mechanical and Tribological Performance of Onyx and Carbon Fiber Composites Produced Through Various Layering Approaches in Continuous Fused Filament Fabricated. J. Inst. Eng. India Ser. D 106, 215–229 (2025).** [**https://doi.org/10.1007/s40033-023-00626-z**](https://doi.org/10.1007/s40033-023-00626-z) **Soundararajan R, Kaviyarasan K, Sathishkumar A, Muthiya Solomon J. Evaluating the impact of post-processing on the wear and friction properties of polyamide 6 carbon fiber composites produced by fused deposition modeling. Proceedings of the Institution of Mechanical Engineers, Part E: Journal of Process Mechanical Engineering. 2024;0(0).** [**https://doi.org/10.1177/09544089241300001**](https://doi.org/10.1177/09544089241300001)

Author response: The recommended references — Soundararajan et al. (2025) and Soundararajan et al. (2024) have been included in the revised manuscript as References 44 and 45, acknowledging their relevance to mechanical and tribological performance of carbon-fiber in polymer composites.
