# Supplementary material for: Effect of graphene addition on tensile, flexural, and hardness behavior of GFRP composites
Source: PLoS One. 2026 Jan 23;21(1):e0340894. doi: 10.1371/journal.pone.0340894 (PMC12829930; doi:10.1371/journal.pone.0340894)
Supplement: Attachment — Submitted filename: Response To Reviewers.docx [file pone.0340894.s004.docx]

**Manuscript ID:** PONE-D-25-37781R1

**Title:** Effect of Graphene Addition on Tensile, Flexural, and Hardness Behaviour of GFRP Composites

# Response to Reviewer #2 – PLOS ONE

**Reviewer -2 Comment:** “Thank you for responding to my comments; however, in the stress–strain curve provided, authors claim that for the 2% and 4% graphene-filled composites there is a noticeable nonlinear region. I do not see any nonlinear behaviour before the ultimate stress point. Please clarify this.”

**Author Response**: We thank the reviewer for raising this point. We agree that the stress–strain curves appear predominantly linear when viewed at the plotted scale. However, a closer examination of the raw tensile data shows a small but measurable reduction in slope for the 2 wt.% and 4 wt.% graphene composites immediately before failure. This subtle deviation from ideal linear elasticity is not visually prominent in the curve due to the limited strain range, but it is supported by SEM evidence indicating early matrix yielding and fiber–matrix debonding.

**Thank you very much for your constrictive comments**
